# Loss of maternal chromosome 11 is a signature event in SDHAF2, SDHD, and VHL-related paragangliomas, but less significant in SDHB-related paragangliomas

**DOI:** 10.18632/oncotarget.14649

**Published:** 2017-01-14

**Authors:** Attje S. Hoekstra, Erik F. Hensen, Ekaterina S. Jordanova, Esther Korpershoek, van der Horst-Schrivers Anouk N.A., Cees Cornelisse, Eleonora P.M. Corssmit, Frederik J. Hes, Jeroen C. Jansen, Henricus P.M. Kunst, Henri J.L.M. Timmers, Adrian Bateman, Diana Eccles, Judith V.M.G. Bovée, Peter Devilee, Jean-Pierre Bayley

**Affiliations:** ^1^ Department of Human Genetics, Leiden University Medical Center, Leiden, The Netherlands; ^2^ Department of Otolaryngology/Head and Neck Surgery, VU University Medical Center, Amsterdam, The Netherlands; ^3^ Department of Pathology, Leiden University Medical Center, Leiden, The Netherlands; ^4^ Department of Pathology, Josephine Nefkens Institute, Erasmus Medical Center Rotterdam, Rotterdam, The Netherlands; ^5^ Department of Endocrinology, University of Groningen, University Medical Center Groningen, Groningen, The Netherlands; ^6^ Department of Endocrinology and Metabolic Diseases, Leiden University Medical Center, Leiden, The Netherlands; ^7^ Department of Clinical Genetics, Leiden University Medical Center, Leiden, The Netherlands; ^8^ Department of Otorhinolaryngology, Leiden University Medical Center, Leiden, The Netherlands; ^9^ Department of Otorhinolaryngology, Head and Neck Surgery, Radboud University Medical Centre, Nijmegen, The Netherlands; ^10^ Department of Medicine, Division of Endocrinology, Radboud University Medical Centre, Nijmegen, The Netherlands; ^11^ Department of Cellular Pathology, University Hospital Southampton, Southampton, UK; ^12^ University of Southampton School of Medicine, Cancer Sciences Division, Somers Cancer Research Building, Southampton, UK

**Keywords:** paraganglioma, pheochromocytoma, succinate dehydrogenase, Von Hippel-Lindau, loss of heterozygosity

## Abstract

Germline mutations in the succinate dehydrogenase (SDHA, SDHB, SDHC, SDHD, SDHAF2) or Von Hippel-Lindau (VHL) genes cause hereditary paraganglioma/pheochromocytoma. While *SDHB* (1p36) and *VHL* (3p25) are associated with autosomal dominant disease, *SDHD* (11q23) and *SDHAF2* (11q13) show a remarkable parent-of-origin effect whereby tumor formation is almost completely dependent on paternal transmission of the mutant allele. Loss of the entire maternal copy of chromosome 11 occurs frequently in *SDHD*-linked tumors, and has been suggested to be the basis for this typical inheritance pattern.

Using fluorescent *in situ* hybridization, microsatellite marker and SNP array analysis, we demonstrate that loss of the entire copy of chromosome 11 is also frequent in *SDHAF2*-related PGLs, occurring in 89% of tumors. Analysis of two imprinted differentially methylated regions (DMR) in 11p15, H19-DMR and KvDMR, showed that this loss always affected the maternal copy of chromosome 11. Likewise, loss of maternal chromosome 11p15 was demonstrated in 85% of SDHD and 75% of *VHL*-related PGLs/PCCs. By contrast, both copies of chromosome 11 were found to be retained in 62% of *SDHB*-mutated PGLs/PCCs, while only 31% showed loss of maternal chromosome 11p15. Genome-wide copy number analysis revealed frequent loss of 1p in *SDHB* mutant tumors and show greater genomic instability compared to *SDHD* and *SDHAF2*.

These results show that loss of the entire copy of maternal chromosome 11 is a highly specific and statistically significant event in *SDHAF2*, *SDHD* and *VHL*-related PGLs/PCCs, but is less significant in *SDHB*-mutated tumors, suggesting that these tumors have a distinct genetic etiology.

## INTRODUCTION

Paragangliomas (PGLs) are neuroendocrine tumors derived from cells of the parasympathetic or sympathetic ganglia. Parasympathetic PGLs occur most commonly in the head and neck region (carotid body, glomus jugulare, and glomus typanicum), are typically benign, and are rarely associated with catecholamine secretion [1, 2]. PGLs arising from the sympathetic ganglia occur in the abdomen and thorax, often secrete catecholamines, and are associated with a higher risk of malignancy. Pheochromocytomas (PCCs) are generally benign paragangliomas that arise in the chromaffin cells of the adrenal medulla, but are frequently associated with hypertension due to excessive catecholamine secretion [3].

Germline mutations in genes encoding subunits of succinate dehydrogenase (SDH, complex II of the mitochondrial respiratory chain) are the most common genetic cause of PGL/PCC, occurring in up to 25% of all cases [4, 5]. SDH is a heterotetramer consisting of two catalytic subunits, SDHA and SDHB, and two membrane-spanning subunits, SDHC and SDHD. SDHAF2 encodes an accessory factor required for the flavination of SDHA. SDH is an essential component of the tricarboxylic acid (TCA) cycle and the mitochondrial respiratory chain. A puzzling aspect of *SDH*x-related disease is that despite the close functional relationship of the SDH proteins, mutations lead to marked differences in both tumor location and clinical phenotype.

Another striking difference is that only mutations in *SDHD* and *SDHAF2*, both located on chromosome 11, show a parent-of-origin inheritance effect in which carriers develop tumors almost exclusively following paternal transmission of the mutation [6, 7]. An important role in causing this inheritance pattern has been ascribed to the loss of the entire *maternal* copy of chromosome 11 in *SDHD*-linked tumors. A cluster of maternally expressed imprinted genes is located on chromosome 11p15, which formed the basis for a hypothesis now known as the ‘Hensen model’. The model proposes that maternal chromosomal 11 loss results in the simultaneous deletion of the *SDHD* wild type gene and an as yet unidentified exclusively maternally expressed gene (or genes), resulting in tumor formation [7]. This hypothesis predicts that loss of the maternal copy of chromosome 11 might be similarly important for *SDHAF2*-linked tumors, but has yet to be demonstrated.

To further clarify the role of loss of the maternal copy of 11p in relation to loss of the long arm of chromosome 11 in paragangliomas, we used several genetic approaches to determine the allelic status of chromosome 11 in *SDHAF2*, *SDHD, SDHB*, and *VHL* mutant PGLs/PCCs. *SDHB* and *VHL*-related tumors were included because these genes map to other chromosomes than 11. The results show that tumorigenesis in *SDHAF2*-related tumors are fully compatible with the Hensen model, and that 11p loss is less important in *SDHB*-related tumors than for the other three tumor subgroups.

## RESULTS

### Loss of heterozygosity in *SDHAF2*-related PGL

**Figure 1:**
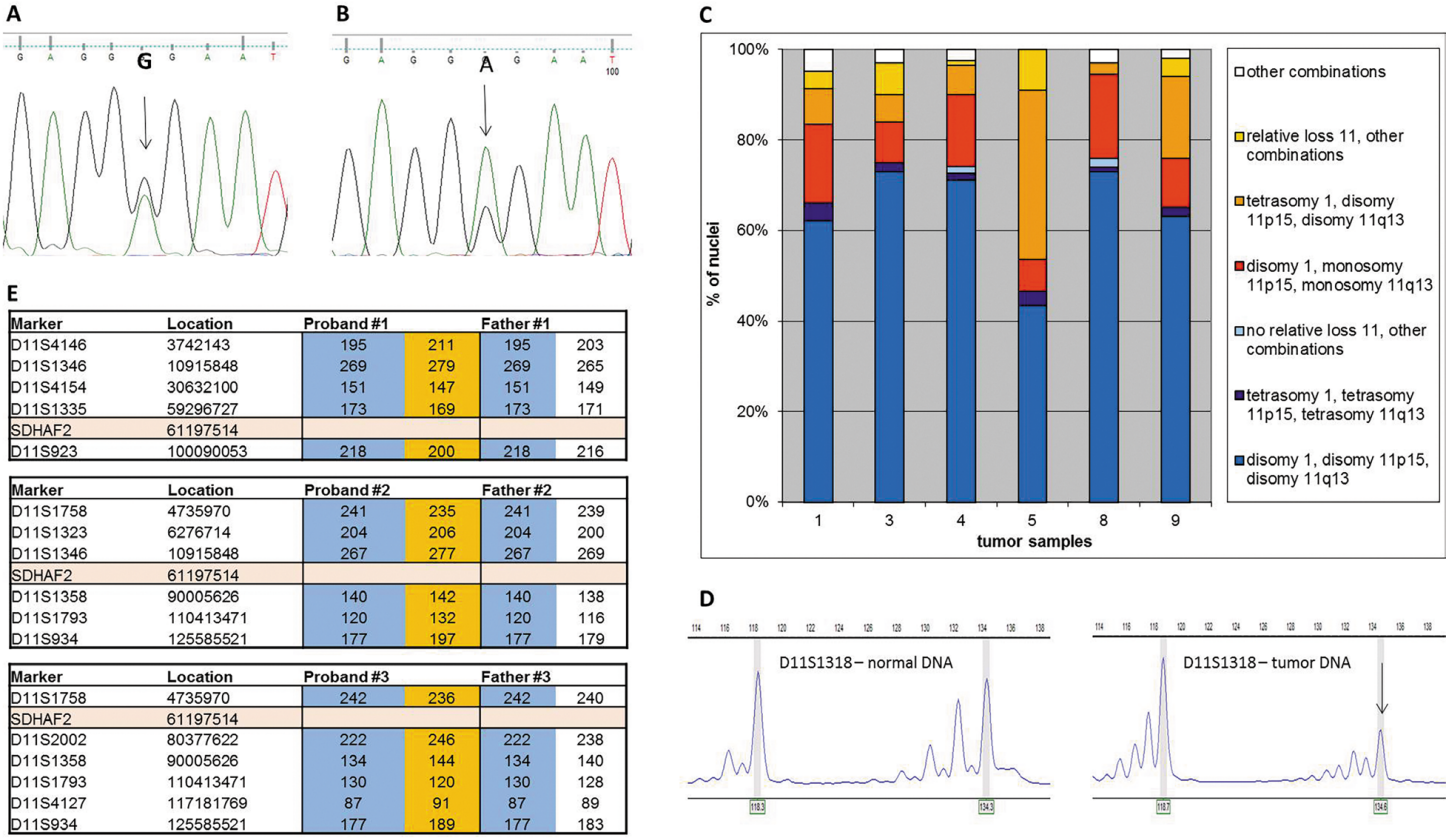
Sanger sequencing of *SDHAF2* in normal (A) and tumor (B) DNA Arrows indicate the relevant nucleotides in the heterozygous patient. (**C**) Interphase FISH results from isolated whole nuclei isolated from paraffin-embedded material of 6 *SDHAF2*-related patients. Frequency distribution of signals obtained with probes for centromere 1 (PUC1.77), telomere 11p (371C18) and telomere 11q (469N6). (**D**) A typical profile of microsatellite marker alleles showing loss of heterozygosity. Arrows indicate the allele lost. (**E**) Chromosome 11 haplotypes of family members. Microsatellite markers are shown with genomic location and the position of *SDHAF2* is indicated. Alleles in orange blocks represent the probable disease haplotype, present in the proband and absent in the father. Alleles in blue blocks represent alleles from the father.

Since *SDHAF2*-related PGLs show a parent-of-origin effect, similar to *SDHD* mutations [7], we hypothesized that their tumorigenesis might also critically depend on loss of the maternal copy of chromosome 11. We first established *SDHAF2* mutation status in germline DNA from 9 patients from *SDHAF2*-related families. All patients showed a missense mutation of *SDHAF2*, c.232G>A (p.Gly78Arg), in a conserved region of the gene [8]. We then sequenced DNA isolated from tumors of all nine patients, and compared this to matched DNA from blood samples. This comparison showed that the wild type allele (guanine (G) nucleotide – arrow, Figure 1A) is underrepresented in tumor DNA (Figure 1B), and the mutant allele is (adenine (A) nucleotide) overrepresented, indicating loss of the wild type allele (loss of heterozygosity - LOH) in the tumor. Partial retention of the wild type allele is characteristic of LOH in PGLs, and is due to admixture with normal cells that proliferate together with tumor cells [9].

### Loss of chromosome 11 in *SDHAF2*-related PGL

To investigate whether the entire copy of chromosome 11 is lost in *SDHAF2*-related tumors, we performed fluorescence *in situ* hybridization (FISH) studies on 6 *SDHAF2*-linked tumors using a probe for the centromere of chromosome 1 as a ploidy reference as described earlier [7], and BAC probes for the subtelomeric regions of 11p and 11q. Simultaneous loss of both probes located on chromosome 11 relative to centromere 1 was found in all *SDHAF2*-related PGLs, in 15–44% of nuclei (Figure 1C, red and orange). Loss of one of the two probes located on chromosome 11 relative to the other was observed in only a very small minority of nuclei (< 0.5%), demonstrating that the observed relative loss involves the entire copy of chromosome 11.

### Parental origin of chromosomal loss in *SDHAF2*-related PGL

To further evaluate LOH in all *SDHAF2*-related tumors and study the parental origin of chromosomal loss, tumor DNA was analyzed for LOH using 24 highly polymorphic microsatellite markers selected from a custom microsatellite database. In the 9 *SDHAF2*-related PGLs investigated, 8 (89%) showed chromosome 11-wide LOH, with allelic imbalance ratios of < 0.7 or >1.3 (Figure 1D, Supplementary Table 2). Parental blood DNA samples were available for 3 *SDHAF2*-related patients. Microsatellite analysis of parental DNA confirmed that the copy of chromosome 11 lost in all 3 *SDHAF2*-mutated tumors was maternal (Figure 1E).

As parental DNA was not available for the remaining cases, we investigated the methylation status of two 11p15.5 DMRs, KvDMR (maternal allele methylated) and H19-DMR (paternal allele methylated). In the presence of two chromosomes with normal methylation levels, each of these DMRs should show an average 50% methylation rate (one chromosome methylated, opposite chromosome unmethylated). This analysis showed hypermethylation of the H19-DMR and hypomethylation in the KvDMR in 8 (89%) *SDHAF2* mutant PGLs (Table 1). These findings are consistent with loss of the maternal allele [10]. The mean methylation rates (± sd) of 7 *SDHAF2*-related tumors differed significantly from the normal methylation rates in matched blood DNA (H19-DMR 0.83 ± 0.13 versus 0.52 ± 0.03 (*p* = 0.008) and KvDMR 0.06 ± 0.07 versus 0.50 ± 0.10 (*p* = 0.006)). In each of the tumors with chromosome 11 loss, the ratio of the methylation rate of H19-DMR/KvDMR was > 3, while the ratio of H19-DMR/KvDMR in blood DNA was ~1. The one *SDHAF2*-related tumor without LOH for chromosome 11 by microsatellite marker analysis, demonstrated normal methylation of both H19-DMR and KvDMR, comparable to blood DNA.

**Table 1:** Methylation status of 11p15 imprinted regions KvDMR and H19-DMR in *SDHAF2* mutant PGLs with and without chromosome 11 LOH

Gene mutation and sample ID	11p15 status	11q12 status	Methylation rate of tumor DNA	H19-DMR/KvDMR methylation rate Ratio of tumor DNA	Methylation rate of matched blood DNA
***SDHAF2 (1)***	LOH	LOH (maternal)	KvDMR: 0.07H19-DMR: 0.57	8.1	
***SDHAF2 (2)***	LOH	LOH	KvDMR: 0.01H19-DMR: 0.94	> 10	KvDMR: 0.59H19 DMR: 0.52
***SDHAF2 (3)***	LOH	LOH	KvDMR: 0.001H19-DMR:0.82	> 10	
***SDHAF2 (4)***	LOH	n.i	KvDMR: 0.03H19-DMR: 0.97	>10	
***SDHAF2 (5)***	LOH	LOH (maternal)	KvDMR: 0.08H19-DMR: 0.79	9.8	KvDMR: 0.47H19 DMR: 0.53
***SDHAF2 (6)***	No LOH	No LOH	KvDMR: 0.63H19-DMR: 0.57	0.9	KvDMR: 0.57H19 DMR: 0.54
***SDHAF2 (7)***	LOH	LOH (maternal)	KvDMR: 0.22H19-DMR: 0.81	3.7	KvDMR: 0.37H19 DMR: 0.47
***SDHAF2 (8)***	LOH	LOH	KvDMR: 0.03H19-DMR: 0.9	> 10	
***SDHAF2 (9)***	LOH	LOH	KvDMR: 0.0001H19-DMR: failed	-	

LOH: loss of heterozygosity. n.i : not informative.

### Frequent loss of maternal chromosome 11 in *SDHD* and *VHL*-related tumors

To investigate the extent and nature of chromosome 11 loss across the various paraganglioma subgroups, we assembled a panel of 26 *SDHD*, 13 *SDHB*, and 8 *VHL*-related PGLs/PCCs. Of the 26 *SDHD*-related tumors investigated using polymorphic microsatellite marker analysis, LOH at all informative markers of chromosome 11 was observed in 22 (85%) tumors (Table 2). In four *SDHD*-related tumors almost all markers showed allele ratios between 0.8 and 1, indicating retention of heterozygosity (Supplementary Table 3 – tumor 23, 26, 27 and 36). Methylation analysis of H19-DMR and KvDMR demonstrated hypermethylation of H19-DMR and hypomethylation of KvDMR in all *SDHD* mutant PGLs with LOH for chromosome 11, consistent with loss of the maternal allele and significantly different from methylation rates of H19-DMR (*p* = 0.004) and KvDMR (*p* = 0.002) in blood DNA (Table 2). In the four *SDHD*-related tumors without chromosomal loss, the ratio of H19-DMR to KvDMR was 1, comparable to blood DNA.

**Table 2:** Methylation status of KvDMR and H19-DMR in *SDHD* mutant PGLs with and without chromosome 11 LOH

Gene mutation and sample ID	11p15 status	11q23 status	KvDMR methylation rate	H19-DMR methylation rate	H19-DMR/KvDMR methylation rate. Ratio
***SDHD (10)***	LOH	LOH	0.01	0.62	> 10
***SDHD (11)***	LOH	LOH	0.0	1.0	> 10
***SDHD (12)***	LOH	LOH	0.01	0.96	>10
***SDHD (13)***	LOH	LOH	0.10	0.86	8.6
***SDHD (14)***	LOH	n.i	0.04	0.97	> 10
***SDHD (15)***	LOH	LOH	0.07	1	> 10
***SDHD (16)***	LOH	n.i	0.02	0.75	>10
***SDHD (17)***	n.i	LOH	0.03	0.45	> 10
***SDHD (18)***	LOH	LOH	0.02	0.96	> 10
***SDHD (19)***	LOH	n.i	0.0	0.91	> 10
***SDHD (20)***	LOH	LOH	0.0	0.87	> 10
***SDHD (21)***	LOH	LOH	0.08	0.76	> 10
***SDHD (22)***	LOH	n.i	0.14	0.90	6.4
***SDHD (23)***	No LOH	No LOH	0.51	0.56	1
***SDHD (24)***	LOH	LOH	0.01	0.7	> 10
***SDHD (25)***	LOH	LOH	0.04	0.86	> 10
***SDHD (26)***	No LOH	No LOH	0.57	0.59	1
***SDHD (27)***	No LOH	No LOH	0.53	0.57	1
***SDHD (28)***	LOH	LOH	0.17	0.66	3.9
***SDHD (29)***	LOH	LOH	0.15	0.68	4.4
***SDHD (30)***	LOH	LOH	0.21	0.70	3.3
***SDHD (31)***	LOH	LOH	0.16	0.68	4.4
***SDHD (32)***	LOH	LOH	0.12	0.71	5.9
***SDHD (33)***	LOH	LOH	0.13	0.65	5.2
***SDHD (34)***	LOH	LOH	0.11	0.70	6.4
***SDHD (35)***	LOH	LOH	0.13	0.69	5.4
***SDHD (36)***	No LOH	No LOH	0.48	0.61	1.2

LOH: loss of heterozygosity. n.i : not informative.

All 8 *VHL*-associated PCCs demonstrated loss of chromosome 11 (Table 3), although in 3 tumors, microsatellite markers were uninformative at 11p15 or at 11q23, while other markers on chromosome 11 showed allelic imbalance ratios of < 0.7 or > 1.3 (Supplementary Table 4).

**Table 3:** Methylation status of KvDMR and H19-DMR in *VHL* mutant PCCs with and without chromosome 11 LOH

Gene mutation and sample ID	11p15 status	11q23 status	KvDMR methylation rate	H19-DMR methylation rate	H19-DMR/KvDMR Methylation rate. Ratio
***VHL (37)***	LOH	n.i	0	0.65	> 10
***VHL (38)***	LOH	LOH	0.01	0.89	> 10
***VHL (39)***	LOH	LOH	0.03	0.75	> 10
***VHL (40)***	LOH	LOH	0.01	1	> 10
***VHL (41)***	LOH	LOH	0.01	1	> 10
***VHL (42)***	LOH	LOH	0.01	0.83	> 10
***VHL (43)***	n.i	LOH	0.01	failed	-
***VHL (44)***	n.i	LOH	0	failed	-

LOH: loss of heterozygosity. n.i : not informative

Methylation status of H19-DMR and KvDMR revealed loss of the maternal copy of chromosome 11 in 6 of 8 (75%) *VHL*-associated PCCs (Table 3), while the methylation status of the H19-DMR could not be determined in 2 (25%) tumors. However, both these tumors showed hypomethylated KvDMR, suggestive of maternal allele loss.

### Low frequency maternal chromosome 11 loss in *SDHB*-related tumors

**Figure 2:**
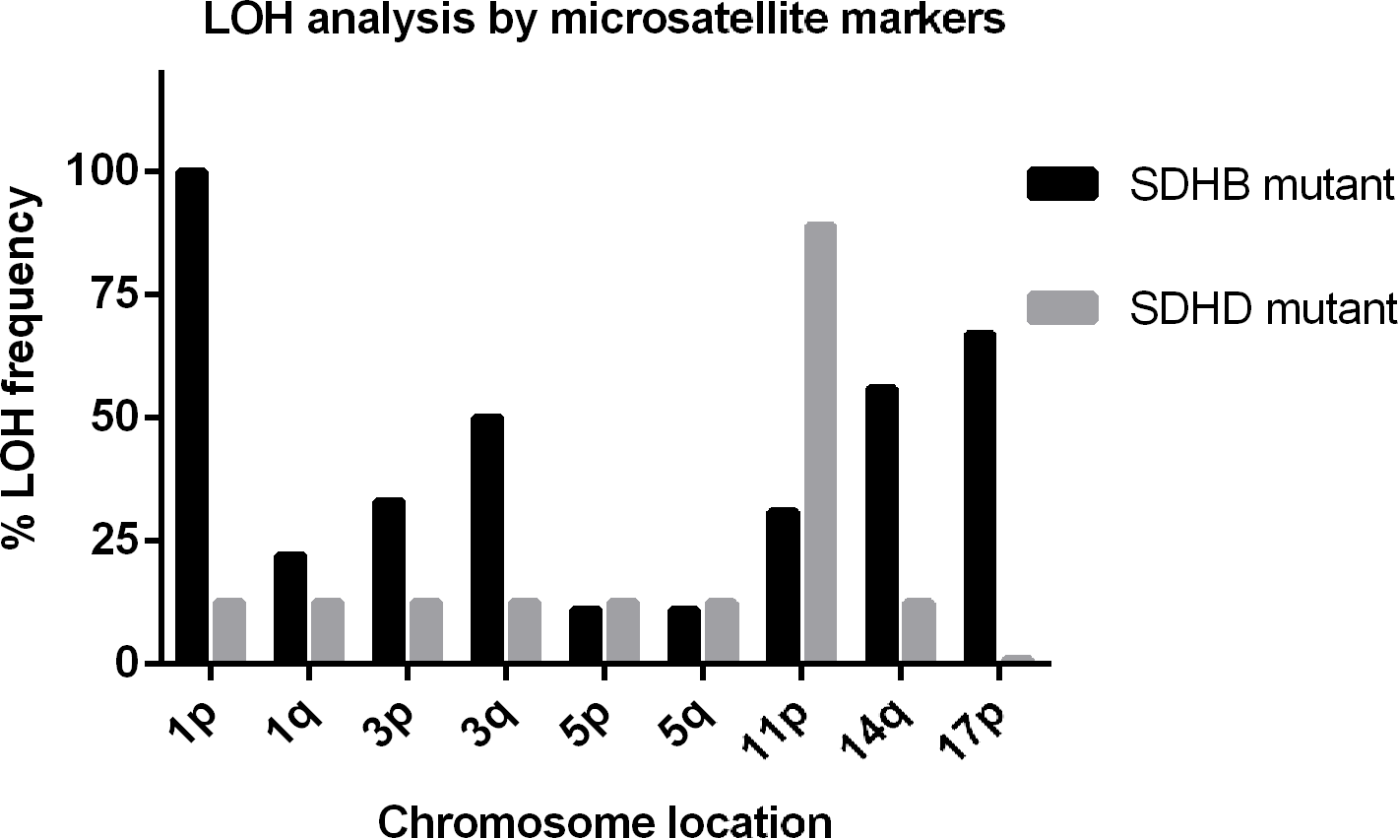
Frequency (%) plot of loss of heterozygosity (LOH) of different chromosomes in *SDHB* (black bars) and *SDHD* (grey bars) mutant tumors, determined by microsatellite marker analysis A higher frequency of LOH of chromosomes 1, 3, 14, and 17 is observed in *SDHB*-related tumors compared to *SDHD*-related tumors. LOH of chromosome 11p is the most frequent event in *SDHD* mutant tumors.

Almost all (92%) *SDHB*-mutated tumors retained heterozygosity in the 11q region, while 4 (31%) tumors showed LOH exclusively in the 11p15 region, in 1 tumor microsatellite markers were uninformative at 11p15 (Table 4). Moreover, in all cases with LOH, this LOH affected multiple small regions of chromosome 11 alternated with regions of retention of heterozygosity (Supplementary Table 5). Methylation analysis of H19-DMR and KvDMR in 8 (62%) *SDHB* mutant tumors showed the ratio of H19-DMR to KvDMR was ~1, comparable to blood DNA. Of the 4 tumors with indications for LOH of 11p15, 1 (tumor 51) showed hypermethylation of H19-DMR and hypomethylation of KvDMR. Tumors 48 and 50 showed hypermethylation of H19-DMR but normal methylation of KvDMR, resulting in a ratio of H19-DMR/KvDMR < 3 (Table 4). In the remaining *SDHB* mutant tumor (tumor 49), no methylation was detected at KvDMR, whereas the methylation rate of H19-DMR was normal. These results are in stark contrast to the unequivocal findings in *SDHAF2*, *SDHD* and *VHL*-related PGLs/PCCs. To investigate whether *SDHB*-mutated tumors show a scattered pattern of LOH on other chromosomes, we used microsatellite markers from chromosomes known to be affected in PGL/PCC [11], including chromosomes 1, 3, 5, 14 and 17. All *SDHB* mutant tumors showed LOH for chromosome 1p, presumably affecting the *SDHB* wild type allele (Figure 2). In addition, LOH of other chromosomes, defined as allelic imbalance ratios of < 0.7 or > 1.3, was observed in most *SDHB*-related tumors, in contrast to *SDHD* mutant tumors (Figure 2, Supplementary Table 6).

**Table 4:** Methylation status of KvDMR and H19-DMR in *SDHB* mutant tumors with and without chromosome 11 LOH

Gene mutation and sample ID	11p15 status	11q23 status	KvDMR methylation rate	H19-DMR methylation rate	H19-DMR/KvDMR methylation rate Ratio	Methylation rate of matched blood DNA
***SDHB (45)***	No LOH	No LOH	0.39	0.45	1.1	KvDMR: 0.39H19: 0.44
***SDHB (46)***	No LOH	No LOH	0.68	0.60	0.9	
***SDHB (47)***	No LOH	No LOH	0.48	0.31	0.6	
***SDHB (48)***	LOH	No LOH	0.57	0.81	1.4	KvDMR: 0.30H19: 0.51
***SDHB (49)***	LOH	No LOH	0.09	0.43	4.8	KvDMR: 0.37H19: 0.51
***SDHB (50)***	LOH	No LOH	0.33	0.91	2.8	
***SDHB (51)***	LOH	LOH	0.04	0.86	> 10	
***SDHB (52)***	n.i	No LOH	0.004	0.47	> 10	
***SDHB (53)***	No LOH	No LOH	0.41	0.69	1.7	
***SDHB (54)***	No LOH	No LOH	0.89	0.97	1.1	KvDMR: 0.50H19: 0.54
***SDHB (55)***	No LOH	No LOH	0.91	0.92	1.0	
***SDHB (56)***	No LOH	No LOH	0.46	0.52	1.1	
***SDHB (57)***	No LOH	No LOH	0.23	0.33	1.4	

LOH: loss of heterozygosity. n.i : not informative

### Greater genomic instability in *SDHB* tumors compared to *SDHD* and *SDHAF2*-related tumors

**Figure 3:**
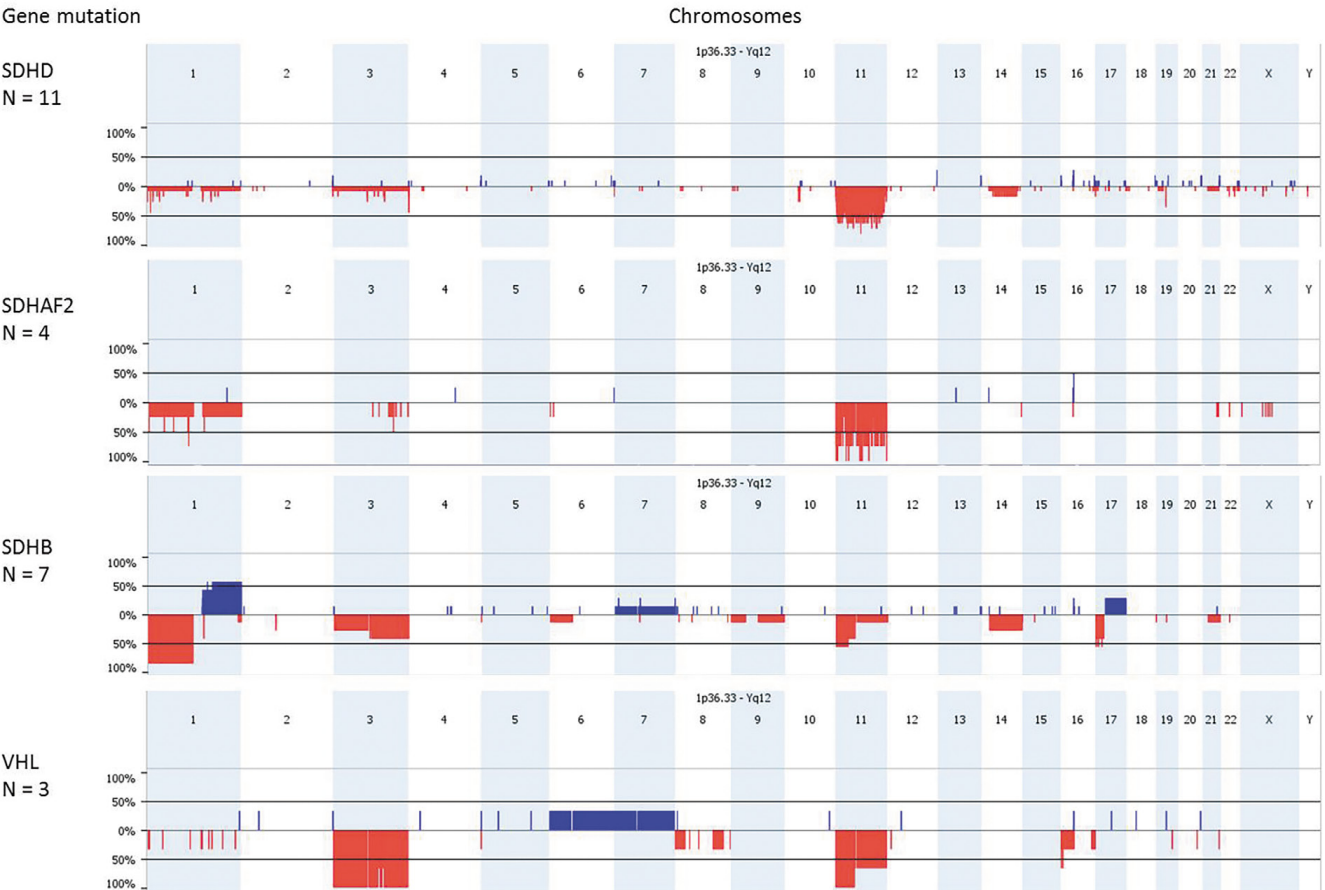
SNP array results of *SDH*x and *VHL*-mutated paragangliomas/pheochromocytomas Genomic frequency plots of gains (in blue) and deletions (in red) among 11 *SDHD*, 4 *SDHAF2*, 7 *SDHB* and 3 *VHL*-mutated tumors, obtained with Nexus Express. *SDHD*, *SDHAF2* and *VHL* mutant paragangliomas/pheochromocytomas have the highest frequency of 11p loss, while the loss of 1p is a frequent event in *SDHB* mutant tumors. The X-axis shows the genomic position along the chromosomes and the Y-axis shows the frequency (%) of copy number gains and losses.

To further explore genomic instability in these tumors, we analyzed genome-wide copy number changes and LOH in a total of 28 tumors (12 *SDHD*, 4 *SDHAF2*, 9 *SDHB* and 3 *VHL*-related PGLs/PCCs) using SNP array analysis. In agreement with our microsatellite marker results and with other studies [11–15], the most frequent copy number alterations in these tumors were deletions of 1p (48%), 3p/q (28%/32%), and 11p/q (88%/68%) (Figure 3). Although the *SDHB*, *VHL*, *SDHD*, and *SDHAF2* genes are located in these chromosomal regions, losses occurred independently of the presence of germline mutations in these genes (Supplementary Figure 2). SNP array analysis revealed patterns of chromosomal gains and losses that were more heterogeneous in *SDHB* mutant tumors compared to *SDHD* and *SDHAF2*-mutated tumors. We evaluated the level of chromosomal instability in each tumor by calculating the ‘Fraction of Aberrant Arms’ (i.e. the proportion of chromosome arms altered over more than 40% of their length [16]). This analysis confirmed the greater degree of genome instability in *SDHB* mutant tumors (mean 12%) compared to *SDHD* mutant tumors (mean 4%) or to *SDHAF2* mutant tumors (mean 4.5%). One *SDHD* and two *SDHB*-linked tumors appeared to be tetraploid as determined by the 2Log (test/reference) ratios (Supplementary Figure 3). The most commonly affected chromosomal regions in *SDHB*-related tumors were gain of 1q (57%), chromosome 7 (28%) and 17q (28%), and loss of 1p (100%) (*SDHB* locus) and 17p (57%). These regions have also been shown to be affected in *RET*, *NF1* and sporadic paragangliomas/pheochromocytomas [11], indicating the potential presence of driver genes on these autosomes.

## DISCUSSION

In this study, we showed that loss of maternal chromosome 11 is also a cardinal feature of *SDHAF2*-linked paragangliomas. The selective loss of maternal chromosome 11 conforms to the Hensen model and explains why *SDHAF2*-linked tumors arise in principal upon paternal transmission of the mutation, comparable to most *SDHD*-linked tumors [7, 12, 17]. The presence of a paternal parent-of-origin effect in both *SDHAF2* and *SDHD*-related PGLs, a phenomenon absent in other *SDH*-related PGLs, argues that their location on chromosome 11 reveals the fundamental role of another chromosome 11 gene in tumorigenesis.

The specific loss of maternal chromosome 11 in *VHL*-related PCCs suggests that a maternally-expressed gene on chromosome 11 also plays a crucial role in these tumors. In this case, no parent-of-origin effect for the *VHL* mutation is predicted because loss of the wild type *VHL* allele on chromosome 3 can occur independently of maternal chromosome 11 loss. These findings agree with earlier reports [10, 11, 14, 18] showing a high frequency of chromosome 11p loss in *VHL* mutant tumors. It is likely that loss of chromosome 11p confers further growth advantage to the tumors besides the inactivation of the *VHL* gene. Interestingly, chromosome 11 loss in *VHL*-related tumors is specific to PGL/PCC and has not been shown in *VHL*-related renal cell carcinomas [19, 20].

The 11p15 region contains several imprinted genes that are exclusively maternally expressed and paternally silenced. LOH of maternal chromosome 11 will result in complete loss of expression of these genes. Our results lend further support to the notion that loss of an as yet unidentified locus (or loci) in 11p15 could contribute to tumor formation in *SDHD, SDHAF2* and *VHL*-related PGLs/PCCs.

Of the potential candidate genes, the paternally expressed growth promoter insulin like growth factor 2 (IGF2) and the maternally expressed candidate tumor suppressor genes *CDKN1C* and *H19*, have been most consistently implicated in imprinting disorders, such as Beckwith-Wiedemann Syndrome and Silver-Russell Syndrome [21]. Interestingly, we recently found loss of *CDKN1C* and *SLC22A18* expression in *SDHD*-related PGLs compared with normal carotid body tissue and established that knockdown of *SDHD* together with *SLC22A18* or with *CDKN1C* led to small increases in cell proliferation and resistance to apoptosis in neuronal cells [22]. Results from the cell line-based functional assays were further supported by the finding that *SDHD* mutant tumors with either retention or loss of chromosome 11 showed equally low levels of SLC22A18 and CDKN1C protein expression [22]. *SDH*x-related tumors are associated with hypermethylation and histone methylation, suggesting a possible mechanism underlying the lowered expression of SLC22A18 and/or CDKN1C in *SDHD* mutant tumors with retention of chromosome 11. Further studies are needed to clarify the role of imprinted genes located on 11p15 in tumor development of *SDHx* and *VHL* mutant PGL/PCC.

While partial or entire loss of chromosome 11 is a signature event in a proportion of *SDHB*-related tumors, many *SDHB* tumors exhibit gains and losses confined to other chromosomes. Compared to *SDHD* and *SDHAF2*, *SDHB* tumors show a more complex pattern of chromosome 11 loss and characteristic changes affecting other chromosomes. Closer analysis to the allele ratios of various microsatellite markers in all tumors revealed a scattered segmental LOH pattern (Supplementary Table 5). While loss might be partly masked by tumor heterogeneity and copy neutral LOH, either of which could impair the detection of genomic alterations [23], the complex pattern of chromosome 11 loss we observed was specific to *SDHB* tumors. Nevertheless, 4 (31%) *SDHB*-related tumors showed loss of maternal chromosome 11p, perhaps signifying a role for the same modifier genes that play such a prominent role in *SDHD* and *SDHAF2* tumors. A low frequency of chromosome 11 loss in *SDHB*-related tumors is in agreement with previous reports [11, 15]. While a proportion of the greater heterogeneity of chromosomal gains and losses we observed in *SDHB* mutant tumors might simply be a byproduct of genomic instability, many changes are recurrent and thus apparently under the influence of selection, especially losses on chromosomes 1p, 3q, 11p, and 17p and somatic gain of chromosome 1q. One or more modifier genes on these autosomes may work in specific synergistic combinations to initiate or promote tumor growth. These recurring and often non-overlapping chromosomal changes also point to a potential redundancy in modifiers, and as such, altered expression of different groups of modifier genes might be involved in *SDHB* tumorigenesis. Analysis of a much larger number of *SDHB* tumors will be required to resolve this question.

Interestingly, recent work [24, 25] showed that engineering *in vitro* the loss of chromosome 8p in cells alters fatty acid and ceramide metabolism. The shift in lipid metabolism triggered tumorigenic potential under stress conditions. Such a complex metabolic shift is difficult to ascribe to a dosage effect of a single gene, and is more likely the result of multiple genes on 8p coordinately undergoing a dosage change. This mechanism might also be at work in *SDH*x-related tumors, with chromosome 11p loss necessary and sufficient to trigger *SDHD* and *SDHAF2* tumorigenesis, whereas *SDHB* tumors require amplification or deletion of multiple driver genes located on different chromosomes.

This speculation is supported by the striking difference in penetrance. A characteristic feature of *SDHD* and *SDHAF2*-related mutations is very high penetrance (90–100%) [8, 26], in contrast to *SDHB* mutations that have an estimated penetrance of only 20–30% [27–29]. This striking difference cannot be readily explained by functional differences between the respective proteins and therefore suggests a role for genetic effects, such as chromosomal location. In this scenario, tumorigenesis in *SDHD* and *SDHAF2* mutation carriers requires only a single somatic genetic event (chromosome 11 loss), as opposed to the two or more independent somatic events required in *SDHB* mutation carriers (loss of the respective wild type allele, together with loss or gain of other chromosomal regions). In conclusion, our results clearly show that *SDHB* tumors follow a more complex and possibly different path to tumorigenesis compared to *SDHD* and *SDHAF2*-related PGLs, involving loss or gain of a greater proportion of the genome.

Despite the apparently integrated function of the SDH subunits, mutations in individual subunit genes result in a number of striking genetic, phenotypic and clinical differences.

Our data now highlight further differences between *SDHB*-related PGL/PCC compared to *SDHD*, *SDHAF2* or *VHL* mutant PGL/PCC in terms of maternal chromosome 11 loss and additional genomic instability. Loss of maternal chromosome 11 is a highly specific and statistically significant event in the latter tumors, suggesting an important role for a still unidentified chromosome 11 factor in the genesis of paraganglioma.

## MATERIALS AND METHODS

### Patients and samples

A total of 44 formalin-fixed, paraffin-embedded (FFPE) tissue samples of PGL/PCC from 41 different patients were used for DNA extraction, including 12 *SDHB*, 16 *SDHD*, 9 *SDHAF2* and 8 *VHL*-related tumors. In addition, we included 12 fresh frozen tumor samples for DNA extraction; 11 *SDHD* and 1 *SDHB*-mutated tumors. The histology of all tumors was reviewed (JVMGB, JPB, ASH) and the mutation detection was confirmed by routine SDHA and SDHB immunohistochemical staining, as described previously [30] (Supplementary Figure 1). For 7 *SDHAF2* mutant tumors and 4 *SDHB* mutant tumors, paired blood lymphocyte DNA samples were available. In addition, parental blood lymphocyte DNA was available for 3 *SDHAF2*-linked patients. Following the original identification of the *SDHAF2* mutation, c.232G>A, p.Gly78Arg, all patients were analyzed by sequencing for the presence of the mutation [8]. The following primers were used for the amplification of exon 2 of the *SDHAF2* gene: 5′-GTTGACCTTCCCAGGCTC-3′ (forward) and 5′-GAGGTTCAGCTGCTTTTCTG-3′ (reverse). Thirty nanograms of genomic DNA from each patient was amplified, and primer annealing was performed at 58°C. PCR fragments were purified using the Nucleospin gel and PCR clean-up kit (Macherey-Nagel). Sequencing was performed using standard protocols. Sequences were analyzed using the Mutation Surveyor software package (Softgenetics).

The *SDHB*-related samples were obtained from Radboud UMC, Nijmegen, The Netherlands, from UMCG, Groningen, The Netherlands and from University Hospital Southampton, UK. *SDHD*-related samples were obtained from the LUMC, Leiden, The Netherlands. *SDHAF2*-related samples were obtained from Radboud UMC and LUMC. *VHL*-related samples were obtained from the Erasmus MC, Rotterdam, The Netherlands. Written informed consent was obtained for DNA testing, further analyses and publication of all results, according to protocols approved by the Ethics Committees of the Erasmus MC, Radboud UMC, and University Hospital Southampton. Tissues from UMCG were used anonymously in accordance with the code for adequate secondary use of tissue, code of conduct: “Proper Secondary Use of Human Tissue” established by the Dutch Federation of Medical Scientific Societies (http://www.federa.org). Oral informed consent was obtained from patients according to protocols approved by the Ethics Committees of the LUMC, Protocol P12.082. Patients’ clinical and genetic data of the tumors included in our study is provided in Supplementary Table 1.

### Triple colour interphase FISH on nuclei isolated from paraffin-embedded tissue

The PUC1.77 probe for the centromeric alphoid repeat DNA of chromosomes 1 was kindly provided by Dr J Wiegant (Department of Molecular Cell Biology, LUMC, Leiden, The Netherlands) [31, 32]. The BAC probes 371C18 (telomere 11p) and 469N6 (telomere 11q) were obtained from the Children's Hospital Oakland Research Institute (Peter de Jong BAC library RP11). All probes were labelled by standard nick translation with biotin-16-aUTP, digoxigenin-11-dUTP or fluorescein-12-dUTP (Roche, Basel, Switzerland).

Isolation of intact nuclei, hybridization and immunodetection were performed as previously described [33], with some modifications. The hybridization mix contained 50% formamide, 3 ng/μl of each of the three probes (either PUC1.77, pLC11A and 3F7 or PUC1.77, 371C18 and 469N6) and a 50-fold excess of human Cot-1 DNA (Invitrogen Life tech., Paisley, UK). A volume of 5 μl of the mix was applied directly onto the slides and covered with an 18 × 18 mm^2^ coverslip. After a denaturation step of 8 min at 80°C, the slides were incubated overnight at 37°C in a moisture chamber. A total of 200 nuclei were analysed for each sample and probe combination by two independent investigators (EFH and ESJ).

### LOH analysis by microsatellite genotyping

Representative tumor areas from FFPE samples were selected to punch 3 cores of 0.6 mm in diameter for DNA isolation. Microdissection was performed on 8 *SDHB*-related tumors, using a total of two 10 μm thick sections for each case. A tumor percentage of greater than 80% was achieved for all tumors. FFPE and fresh frozen tumor samples were incubated overnight with proteinase K at 60°C and DNA was isolated using the Qiagen FFPE DNA kit or QIAamp DNA Mini Kit (Qiagen Benelux B.V., Venlo, The Netherlands), respectively, according to the manufacturer's instructions. Tumor and blood samples were genotyped for microsatellite markers located on chromosome 11, as described in the results section. Primer sequences of the microsatellite markers are described in Supplementary Table 7. For each marker, 40 ng of DNA was amplified over 40 cycles using FastStar Taq DNA Polymerase (Roche). Forward primers were labeled with 6-FAM, HEX or NED fluorophore (Sigma-Aldrich, St. Louis, MO, USA). Amplicons of microsatellite markers were run on an ABI 3730 genetic analyzer and data were analyzed using Gene Marker software (Soft Genetics, State College, PA 16803, USA), using ABI GeneScan Rox 400 as the internal size standards. LOH was calculated using the allelic imbalance ratio: AIR = (Tumor1/Tumor2)/(Normal1/Normal2). Tumors were regarded as positive for LOH when the mean allele ratio between tumor and blood was < 0.7 for all informative markers, as described earlier [34]. In cases where no matching blood lymphocyte DNA sample was available, allele peak ratios were compared to DNA samples with the same or very similar allele combinations. Some markers were either not informative in the patient or did not perform well enough on tumor DNA samples to give a reliable result and were therefore excluded.

### Methylation analysis of H19-DMR and KvDMR

Bisulfite conversion of 250 ng of tumor DNA was performed with the EZ DNA Methylation Kit (Zymo Research, Irvine, CA, USA) according to the manufacturer's instructions. Bisulfite treated DNA was amplified by PCR with primers specific for modified DNA, designed using Methprimer [35]. Primer sequences for the H19-DMR were 5′-GGTTT TAGTGTGAAATTTTTTT-3′ (forward) and 5′-CCATAAATATCCTATTCCCAAATAAC-3′ (reverse) and for the KvDMR 5′-TTGAGGAGTTTTTTGGAGGTT-3′ (forward) and 5′-ACCC AACCAATACCTCATAC-3′ (reverse). The PCR program consisted of an initial denaturation step at 94°C for 15 minutes followed by 44 cycles of 20 seconds at 94°C, 30 seconds at 55°C for the KvDMR and 52.5°C for the H19-DMR, followed by 5 minutes at 72°C. PCR fragments were purified using the Nucleospin gel and PCR clean-up kit (Macherey-Nagel, Düren, Germany). Sanger sequencing was performed using standard protocols and methylation rates were evaluated using ESME software [36].

### Oncoscan analysis

Twelve *SDHD*, four *SDHAF2*, nine *SDHB*, and three *VHL* mutant tumors (Supplementary Table 1) were further investigated for whole genome copy number by OncoScan analysis (molecular inversion probe technology), as described in [37]. This array consists of ~335.000 probes of which the majority (~283.000) are SNP-based. DNA was processed by the Affymetrix Research Services Laboratory (Santa Clara, California, USA) using the OncoScan™ FFPE Assay. The normalized OncoScan data (2Log (test/reference)-ratios and B-allele frequency plots) were analyzed with the Nexus Express software version 3.1 (Biodiscovery, Inc, El Segundo, California, USA) for copy number calling.

### Statistical analysis

IBM SPSS Statistics 20.0 for Windows software package (SPSS, Armonk, NY: IBM Corp) was used to analyze the results. Statistical significance between two groups was determined by Mann-Whitney *U* test. *P* < 0.05 was considered statistically significant.

## SUPPLEMENTARY MATERIALS FIGURES AND TABLES














