# Supplementary material for: Loss of maternal chromosome 11 is a signature event in SDHAF2, SDHD, and VHL-related paragangliomas, but less significant in SDHB-related paragangliomas
Source: Oncotarget. 2017 Jan 14;8(9):14525–36. doi: 10.18632/oncotarget.14649 (PMC5362423; doi:10.18632/oncotarget.14649)
Supplement: Supplementary file 1 [file oncotarget-08-14525-s001.pdf]

Loss of maternal chromosome 11 is a signature event in *SDHAF2*, *SDHD*, and *VHL*-related paragangliomas, but less significant in *SDHB*-related paragangliomas

Supplementary Materials

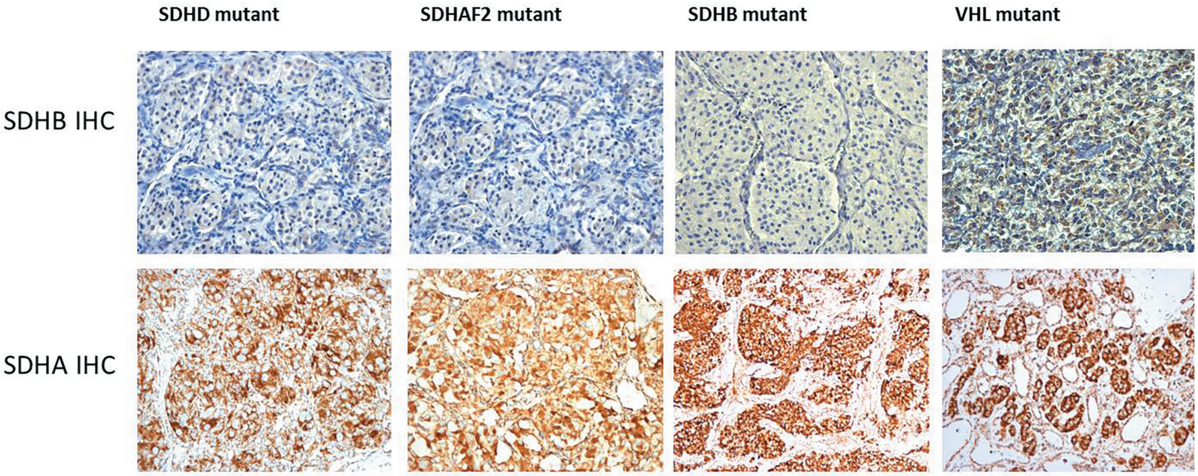

Supplementary Figure 1: Immunohistochemical (IHC) staining of *SDHx* and *VHL*-mutated paragangliomas/ pheochromocytomas. SDHB protein expression is negative in *SDHx*-related tumors and positive in *VHL* mutant tumors. SDHA protein expression is positive in *SDHD, SDHAF2, SDHB* and *VHL*-mutated tumors.

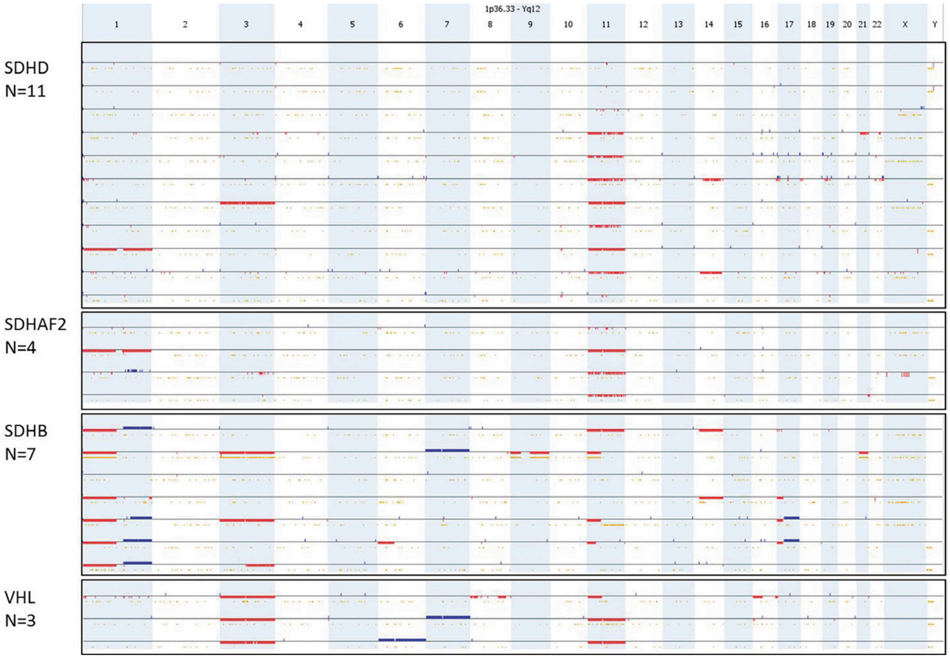

Supplementary Figure 2: Overview of the copy-number profiles exported from Nexus Express. Each line represents the profile of a tumor, with gains in blue and deletions in red. Samples are ordered according to the gene mutation, as indicated on the left.

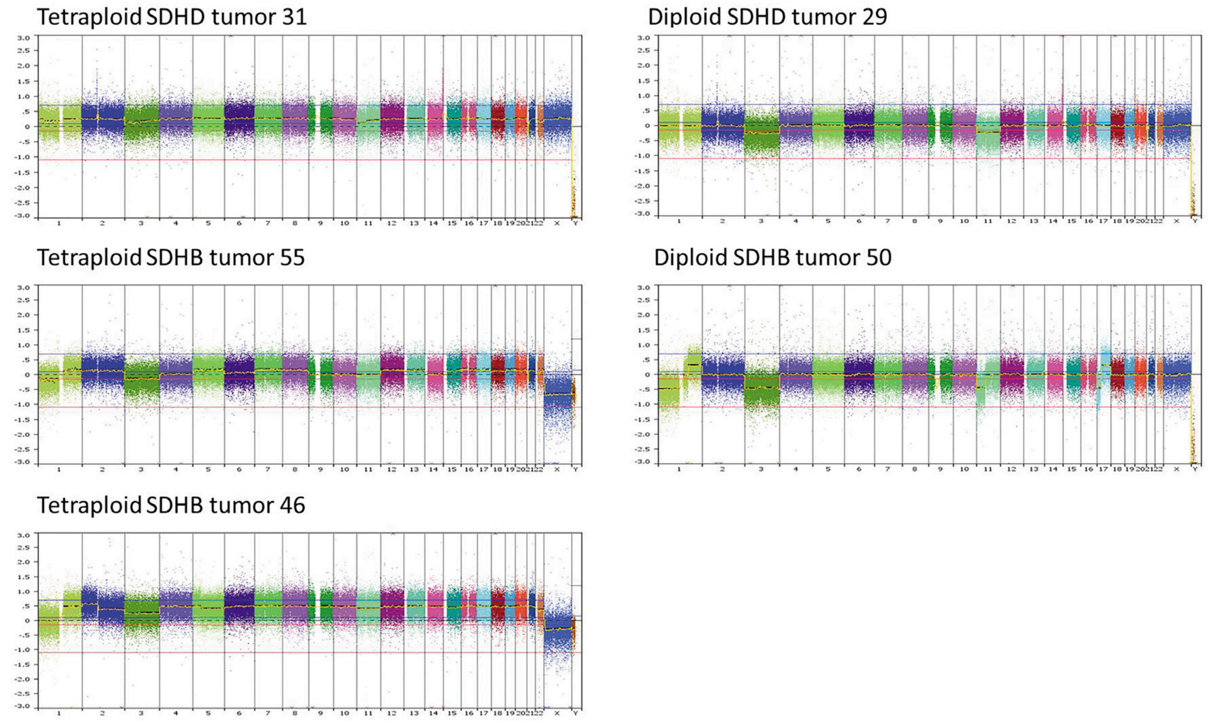

Supplementary Figure 3: Log Ratio profiles of five different tumors. The left panel represents three different tetraploid tumors from one *SDHD* (average log ratio 0.3) and two *SDHB* mutation carriers (tumor 55, average log ratio 0.25; tumor 46, average log ratio 0.5). For comparison, the right panel represents two diploid tumors from one *SDHD* and one *SDHB* mutation carrier, both with an average log ratio of 0.

Supplementary Table 1: Clinical and genomic characteristics of PGL/PCC tumors. See Supplementary_Table_1

Supplementary Table 2: Microsatellite loss of heterozygosity analysis of chromosome 11 and methylation status analysis of the imprinted regions KvDMR and H19-DMR in *SDHAF2* mutant PGLs. See Supplementary_Table_2

Supplementary Table 3: Microsatellite loss of heterozygosity analysis of chromosome 11 and methylation status analysis of the imprinted regions KvDMR and H19-DMR in *SDHD* mutant PGLs. See Supplementary_Table_3

Supplementary Table 4: Microsatellite loss of heterozygosity analysis of chromosome 11 and methylation status analysis of the imprinted regions KvDMR and H19-DMR in *VHL* mutant PCCs. See Supplementary_Table_4

Supplementary Table 5: Microsatellite loss of heterozygosity analysis of chromosome 11 and methylation status analysis of the imprinted regions KvDMR and H19-DMR in *SDHB* mutant PGL/PCCs. See Supplementary_Table_5

Supplementary Table 6: Microsatellite loss of heterozygosity analysis of selected chromosomes in *SDHB* and *SDHD* mutant tumors. See Supplementary_Table_6

Supplementary Table 7 : Primer sequences of chromosome 11 microsatellite markers

Microsatellite marker	Primer sequence (forward)	Primer sequence (reverse)
D11S1363	GAAAATGGTATTTAGAAACCAA	CCCAAGGGCTTACAAC
D11S4177	GACGGTGAACCCAGTCATT	TGTCAGCACAGAGGCAGAGT
D11S1318	CCCGTATGGCAACAGG	TGTGCATGTACATGAGTG
D11S4146	AACACGAGGTTAAGCAGAG	GAATGAAGAATTTTCCAAACTAC
D11S1758	GTCAAGGTAGCCCAGGAAAT	CCAGTGTTCTCACAAACTGAGTA
D11S1760	GATCTCAAGTGTTTCCCCAC	AAACGATGTCTGTCCACTCA
D11S1323	TGCTGCTTAGAATGAGTAGATGTC	CTCTATGAAGTTGGAGTCTAGGTTG
D11S932	TCGTATAGCACAACCTTGGC	CTTATCATCTCTGGGTAGTGAAGTC
D11S4149	TGAATTATACCCCTGACCAA	CCCAGCCAATATCAGCA
D11S1346	GCCAAATTAAGAGGCG	CCCAGGGTTGTTGTGA
D11S4154	ATCCCTTGGCTTTCTCAGAGCAC	GGTGCCCCTAACCTCCATGT
D11S4174	GATTAAATGCCCACTATGTAGC	GATAGCTTTCCCAGATGGTT
D11S1335	CACAATCCTATGAAGCAGGT	CTACCAATTGCTGGGTTG
D11S1253	CCAGAGCAGCTCAAATATGAAAACTGAGGA	GCTTACAGTTTACATCATCTGC
D11S4076	CATGAATGCTCTTGTCCC	AACCCCCTGGAAAATAGACT
D11S4205	GATAGATACGTTAATTTGCTTCACC	GCTTAGTTGCTATTACATGATGACC
D11S913	CATTTGGGAAATCCAGAAGA	TAGGTGTCTTATTTTTTGTTGCTTC
D11S2002	CATGGCCCTTCTTTTCATAG	AATGAGGTCTTACTTTGTTGCC
D11S1358	ACAACCTGGATGAACCC	ACTTTCTGTCTTTATGATTTTGATT
D11S923	ATCTATAATATCTGGAAGGTACTGG	ATTAGGGCTGGATTGAGG
D11S1793	AGTCATGCATCCTCCCTGTA	ATCCTGAACACATTCCTCAA
D11S4127	ATGAGAAGTGCCATCCAGC	ACTATGCCCAGTGTGTGTGC
D11S934	GCTGTCCCTGACAACTACATGC	TTCCATCAGAACTGGGAATGAG
D11S4098	TCCCAGGAACATCAGC	TTGCATTTCACCAAAAGA
